# Hyaluronic acid modified carbon nanotubes using for photothermal therapy by promoting apoptosis of nasopharyngeal carcinoma cells

**DOI:** 10.3389/fbioe.2023.1229852

**Published:** 2023-07-04

**Authors:** Zeyu Guo, Xianzhi Liu, Yiyi Lin, Zelin Sang, Dong Chen

**Affiliations:** ^1^ The First Affiliated Hospital of Jinzhou Medical University, Jinzhou, China; ^2^ Jinzhou Medical University, Jinzhou, China

**Keywords:** carbon nanotubes, hyaluronic acid, photothermal therapy, nasopharyngeal carcinoma, apoptosis

## Abstract

**Background:** The present work illustrates the role of multi-walled carbon nanotubes in photothermal therapy. Nasopharyngeal carcinoma (NPC) is a malignant tumor of the head and neck with significant ethnic and geographic differences, and conventional treatment options are no longer suitable to improve the prognosis and survival of patients. Photothermal therapy (PTT) has emerged as a new strategy for oncology treatment in recent years and is now used in the treatment of many common cancers. Multi-walled carbon nanotubes (MWCNT) have been used to advantage in several fields due to their excellent thermal conductivity. The aim of this paper is to investigate the promotion of apoptosis of nasopharyngeal cancer cells by multi-walled carbon nanotubes as an adjuvant nanomaterial for nasopharyngeal cancer photothermal therapy.

**Methods:** Carboxylated multi-walled carbon nanotubes and prepared multi-walled carbon nanotube-hyaluronic acid (MWCNT-HA) composites were used for cell proliferation-related experiments such as CCK-8 assay, live-dead staining and flow cytometric analysis and inverted fluorescence microscopy to determine the expression level of apoptotic factors and confocal microscopy cell morphology analysis on nasopharyngeal carcinoma CNE-1 cells under near-infrared laser irradiation. The effects of multi-walled carbon nanotubes on the proliferation and apoptosis of tumor cells under NIR response were elucidated, and the mechanism of apoptosis was explored.

**Results:** TEM and SEM demonstrated that MWCNT had good appearance morphology and the temperature rise curve indicated excellent photothermal stability. And MWCNT and MWCNT-HA could significantly inhibit the proliferation of tumor cells and change the normal morphology of cells under NIR laser irradiation. Cellular immunofluorescence analysis confirmed that MWCNT-HA significantly upregulated the expression level of apoptosis factor Caspase-3 and significantly downregulated the expression level of anti-apoptosis factor Bcl-2.

**Conclusion:** In this study, MWCNT inhibited the proliferation of tumor cells and promoted apoptosis through the use of multi-walled carbon nanotubes as an adjuvant nanomaterial for photothermal therapy. In addition, multi-walled carbon nanotubes could inhibit the mitochondrial pathway of CNE-1 cells to cause cell death. These studies suggest that multi-walled carbon nanotubes can function as efficient photothermal conversion materials for tumor photothermal therapy.

## 1 Introduction

Nasopharyngeal carcinoma (NPC) is a common malignant tumor of the head and neck with significant ethnic and geographic distribution differences, with a high incidence in yellow populations and more common in southern China and Southeast Asia (Chang et al., 2021). The incidence and mortality rates in China are among the highest in the world (Chua et al., 2016). Currently, the main treatment options include surgical resection, systemic chemotherapy, and local radiotherapy. However, due to the disease characteristics of nasopharyngeal carcinoma, which is prone to recurrence, delayed diagnosis due to early metastasis, and toxic effects from long-term radiotherapy, it may lead to a serious reduction in patients’ quality of life (Chua et al., 2016; Haleshappa et al., 2017; Xu et al., 2017). Therefore, it is of great importance to find a treatment with lower side effects on the human body.

In recent years, with the rapid development of medical technology, photothermal therapy (PTT) has gradually become a new tumor treatment option, which is attracting attention (Ma and Xue, 2021). Compared with traditional treatment options, photothermal therapy has the advantages of good tissue permeability and low invasiveness, and nanomaterials play a key role in photothermal therapy as an important adjunct to photothermal therapy. Among various types of nanomaterials, inorganic nanomaterials with strong near-infrared laser irradiation (NIR) absorption can be used as highly effective photothermal agents for cancer and bacterial infections due to their high specific surface area and unique photoelectric properties, and nanomaterials-assisted photothermal therapy has great application potential. Among the many inorganic nanomaterials that have been proved as an adjunct to photothermal therapy, carbon-based materials are more excellent materials used to mediate the local photothermal effect of tumors (Fernandes et al., 2020; Liu et al., 2020), and multi-walled carbon nanotubes are included as a common carbon-based material. It has superior structural and physical properties, due to its high aspect ratio, chemical inertness, high strength, and high electrical and thermal conductivity and stability of photothermal conversion when suspended in aqueous, inorganic or organic solutions, and has been widely used in many fields, such as environmental testing, pharmacology, food and forensic chemistry (Kartel et al., 2016; Oliveira and Morais, 2018; Dragan et al., 2021), and also in the field of new energy catalysis due to its excellent physical properties and affordable production cost. They also play an important role in the field of new energy catalysis due to their excellent physical properties and affordable production cost (El Attar et al., 2022). However, the role of multi-walled carbon nanotubes in the biomedical field is questionable, as some animal experiments have shown that multi-walled carbon nanotubes deposited in the lungs after prolonged inhalation in exposed environments in large quantities can lead to an increased risk of inflammation, fibrosis and granulomatous lesions in the lungs and cause mutations in certain genes (Porter et al., 2010; Sager et al., 2022). It has also been shown that multi-walled carbon nanotubes can improve the efficacy in the treatment of breast cancer and can be used as a new method to improve the accuracy of breast cancer diagnosis (Dizaji et al., 2020). The good biocompatibility of hyaluronic acid (HA), a negatively charged linear mucopolysaccharide widely found in vertebrate tissues, can accommodate the specificity of multi-walled carbon nanotubes, which are specially treated and engineered to achieve the desired therapeutic effect. The use of polysaccharide-modified multi-walled carbon nanotubes can effectively improve biosafety and compatibility, and have been used as carriers carrying targeted drugs in the treatment of malignant tumors such as lung cancer and colon cancer, showing excellent drug release and absorption efficiency and strengthening the therapeutic effect on tumors (Singh et al., 2017; Prajapati et al., 2019). In this context, we report the therapeutic effect of MWCNT for NPC under photothermal therapy.

In this paper, in order to reach the function of NIR laser response and reduce the proliferation of tumor cells, we prepared multi-walled carbon nanotube-hyaluronic acid (MWCNT-HA) combined composites and as efficient photothermal conversion materials in photothermal therapy to assist NIR laser irradiation for antitumor treatment. In our experiments, we found that MWCNT and MWCNT-HA could effectively inhibit the proliferation rate of nasopharyngeal carcinoma CNE-1 cells by photothermal therapy and enhance the induction of CNE-1 cell apoptosis and alter the cell morphology of CNE-1 cells by promoting the expression of the tumor-associated apoptosis factor Caspase-3 and inhibiting Bcl-2. This study provides valuable information on NIR-responsive MWCNT and MWCNT-HA materials as efficient inorganic nanomaterials for photothermal therapy of nasopharyngeal carcinoma.

## 2 Materials and methods

### 2.1 Materials

Primary antibodies to Bcl-2, caspase-3, and *β*-microtubulin were purchased from Cell Signaling Technology (United States). The secondary antibodies to HRP AffinPure goat anti-rabbit IgG and HRP AffinPure goat anti-mouse IgG were purchased from Proteintech (United States). The Alexa Fluor^®^488 goat anti-mouse/rabbit IgG and Alexa Fluor^®^568 goat anti-mouse/rabbit IgG were purchased from Invitrogen (United States). Triton X-100 and 4,6-dimethyl-2-phenylindole (DAPI) were purchased from Abcam (United Kingdom).

### 2.2 Cell lines and treatments

Nasopharyngeal carcinoma (NPC) CNE-1 cell line was obtained from Chunmai Biotechnology Co. and cultured in RPMI-1640 medium (Corning, Manassas, VA, United States) with 10% FBS (Gibco, New York, United States) and 1% penicillin/streptomycin in a humidified incubator with 5% CO_2_ at 37°C. Cells were passaged every 3 days at a 1:2 ratio. Cells at the exponential growth stage were used for the experiments, and the experimental groups were divided into 3 groups: control group (blank group), MWCNT photothermal reaction experimental group and MWCNT-HA photothermal reaction experimental group.

### 2.3 Preparation of MWCNT-HA

Carboxylated multi-walled carbon nanotubes were purchased from Guoheng Qixiang Technology Co. Their thickness was 10–20 nm. before use, we suspended them in distilled water for 1 h using ultrasound, and then passed them through a centrifugal filtration device (10 kDa MWCO, Millipore Amicon) for initial filtration, followed by extensive washing to further purify the raw material. This was followed by filtration and washing in the above manner using methanol.

Acid treatment of MWCNT: 250 mg MWCNT was immersed in a mixture of concentrated sulfuric acid (H_2_SO_4_) and nitric acid (HNO_3_) (mixed in a 3:1 ratio by volume) in a flat-bottomed flask and refluxed at 100 rpm for 24 h at 120°C ± 2°C under constant magnetic stirring. The treated MWCNT was then rinsed with deionized water and ultra-centrifuged at 20,000 rpm for 15 min, followed by vacuum drying.

50 mg of HA was dissolved in distilled water at pH 8.0 and activated with 0.50 mM N-hydroxysuccinimide and 0.50 mM EDC. HCl for 6 h. This activated HA solution was added to the carboxylated MWCNT aqueous suspension separately. Subsequently, MWCNT-HA was separated by dialysis and dried in the open air for 12 h (Prajapati et al., 2019). The final MWCNT-HA composites were obtained.

### 2.4 Characterization

Transmission electron microscopy (TEM, JEM-1200EX, Tokyo, Japan) was used to observe the appearance of MWCNT. The morphological characteristics of MWCNT were examined using a scanning electron microscope (SEM, Gemini300, Berlin, Germany). The composition of MWCNT, MWCNT-HA was determined by Fourier transform infrared spectroscopy (FTIR, SHIMADZU, Tokyo, Japan) using the potassium bromide disc method. The crystal structure of MWCNT was tested by X-ray diffraction (XRD, SHIMADZU, Tokyo, Japan). The NIR near infrared laser was purchased from Busan Electronics Co.

### 2.5 Cell viability assay

The CCK-8 method was used for the assay. Cells were cultured in three 96-well plates (5×10^3^/well), and each well was divided into three groups: blank, MWCNT, and MWCNT-HA. The cells in each well and 50 μL of culture medium were added to the wells for cell culture, and to the MWCNT and MWCNT-HA experimental groups were added 50 μg/mL MWCNT, MWCNT-HA suspension and laser irradiated for 2 h. Then, the cells were incubated for day1, day2, day3.30 μL of CCK-8 solution purchased from KeyGEN BioTECH (Jiangsu, China) was added to each well and the cells were incubated at 37°C for 2 h. The absorbance at 450 nm was measured at day1, day2 and day3 for the blank group and the treated group respectively using a microplate reader. Finally, the OD value was calculated using the formula.

The CNE-1 cells were incubated with MWCNT (50 μg/mL) or MWCNT-HA (50 μg/mL) for 24 h. Afterwards, they were treated with NIR laser for 5 min, using the NIR laser from Beyotime Biotechnology (Shanghai, China) purchased from Calcein AM/PI mixed fluorescent dye was used to stain the cells, and after treatment, the cells were incubated at 37°C for 30 min to label live versus dead cells. Finally, live/dead cells were imaged using fluorescence microscopy for the blank group versus the experimental group.

### 2.6 Flow cytometry analysis

CNE-1 cells were inoculated in 6-well plates with approximately 1 × 10^5^ per well cells and cultured for 24 h. We next incubated CNE-1 cells with MWCNT or MWCNT-HA ((50 μg/mL) for 12 h. The treated CNE-1 cells were treated with 808 nm NIR laser for 5 min 12 h after the previous treatment step, and the cells were collected. We studied apoptosis by flow cytometry analysis using the modulin-FITC/PI apoptosis kit, strictly following the manufacturer’s instructions.

### 2.7 Cell immunofluorescence staining

Cells were cultured in 3 wells of 2 6-well plates (1 × 10^5^/well), divided into two experimental groups of blank and MWCNT-HA adding the appropriate amount of MWCNT-HA (50 μg/mL), incubated for 24 h, and then irradiated uniformly for 5 min using a NIR laser. After that, the original culture medium was aspirated, the wells in the 6-well plate were washed twice with PBS, cell fixative was added for 40 min to fix the cells, the fixative was aspirated, PBS was washed 3 times, then permeabilized with 0.1% Triton X-100 for 20 min, Triton was aspirated, PBS was washed 3 times, closed with normal goat serum for 2 h, after that, PBS was washed 3 times again, and anti-tubulin (1: 500), anti-caspase-3 (1: 500) and anti-bcl-2 (1: 500), and incubated overnight at 4°C. The next day add Alexa Fluor^®^ 568 goat anti-mouse/rabbit IgG (1: 1,000) and Alexa Fluor^®^ 488 goat anti-mouse/rabbit IgG and incubate for 2 h at room temperature protected from light. Cells were washed 3–5 times with 0.1% PBS (3–5 min) and incubated for 15 min using DAPI. Finally, the above treated cells were observed using an inverted fluorescence microscope.

### 2.8 Cell morphology confocal assay

The treatments and procedures were approximately the same as for the apoptotic factor assay, in brief, CNE-1 cells were cultured in a confocal dish. The cells were washed three times with PBS and then permeabilized with 0.1% Triton X-100 for 30 min. Cells were blocked with normal goat serum for 2 h and then washed three times with 0.1% PBS. Anti-tubulin (1: 500) was added and incubated overnight at 4°C. The next day add Alexa Fluor^®^ 568 goat anti-mouse/rabbit IgG (1: 1,000) and Alexa Fluor^®^ 488 goat anti-mouse/rabbit IgG and incubate for 2 h at room temperature protected from light. Cells were rinsed with 0.1% PBS (3–5 min) 3–5 times and incubated with DAPI for 15 min. Finally, cell morphology was observed using a high-resolution confocal microscope.

### 2.9 Statistical analysis

Statistical analysis of all collected data was performed using SPSS 19.0 and expressed as mean standard deviation (SD). One-way analysis of variance (ANOVA) was used to compare the two groups. Statistical significance was defined as a *p*-value less than 0.05.

## 3 Results

### 3.1 Characterization of the MWCNT and MWCNT-HA

**FIGURE 1:**
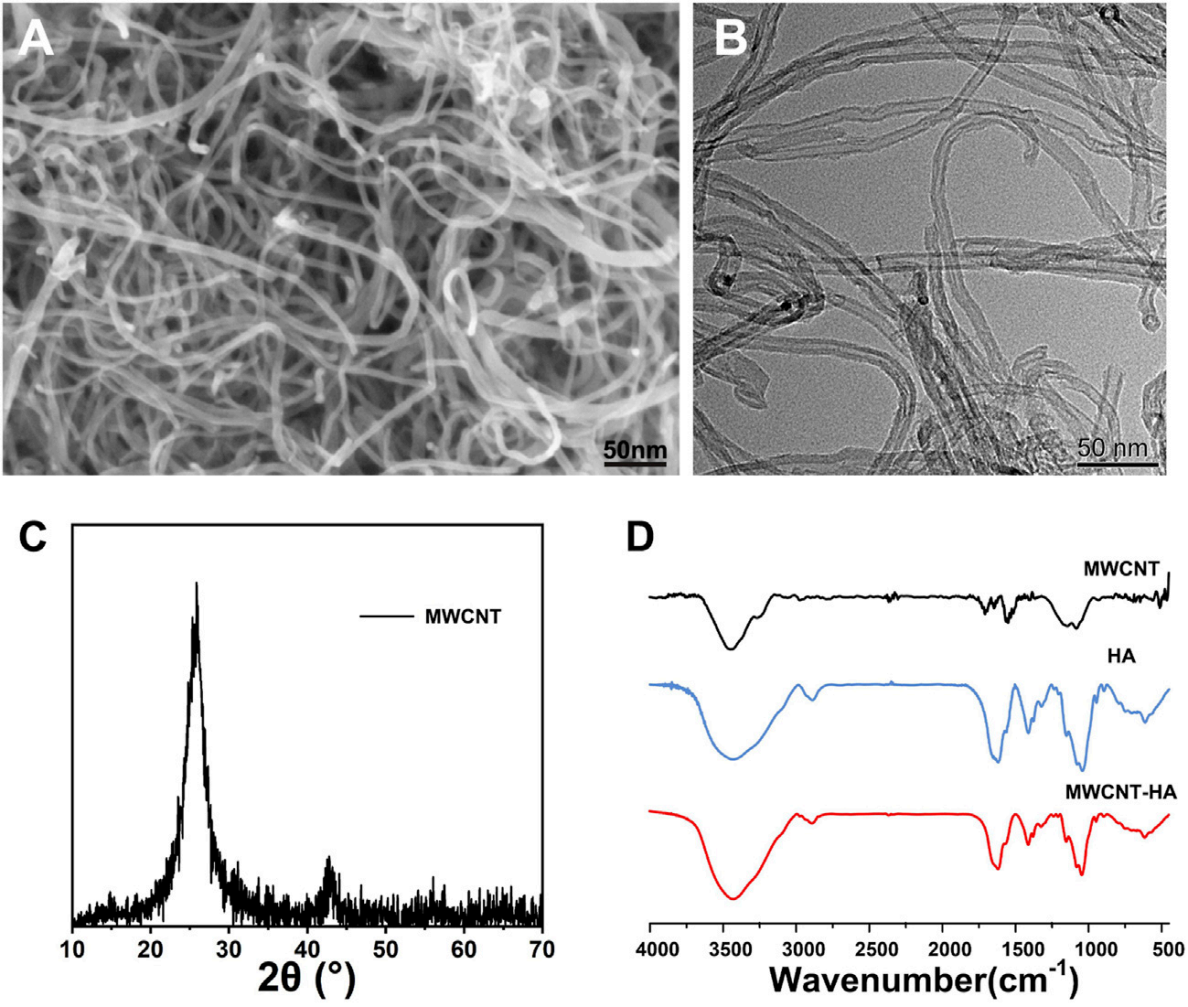
Characterization of MWCNTs and MWCNT-HAs. **(A)**The SEM images of MWCNT and **(B)**The TEM images of MWCNT. **(C)**The XRD images of MWCNT **(D)**The FTIR spectra of MWCNT, HA, MWCNT-HA.

The morphologies of purified MWCNTs were characterized using SEM and TEM, respectively, Figure 1A in which we observed the surface morphology of MWCNT using SEM and found that the multi-walled carbon nanotubes have 1D structure. As shown in Figure 1B, we observed the shape and surface morphology of MWCNT by TEM, and the purified MWCNT showed open ends under the transmission electron microscopy photograph, and it was seen from the figure that it was hollow tubular structure with nanometer size. The ends of the MWCNTs in Figures 1A,B have irregularly shaped nodules, which are carbon particle impurities in the unpurified MWCNT. According to Figure 1C, which shows the XRD spectrum of MWCNT, we found a sharp characteristic peak at 2θ = 26.138°. And a lower intensity peak was observed at 2θ = 42.871° and which confirmed the presence of pure carbon nanotubes. FTIR spectra were used to determine the functional groups of MWCNT, HA, and the synthesized complexes. Deeper peaks in frequency indicate functional groups with lower absorption of incident light, while higher absorption peaks represent functional groups with higher vibrational energies comparable to the radiant light energy. Based on FTIR spectra, we found that for HA, at 1,100 cm^-1^ is the characteristic peak of C=O, and for purified MWCNT at 1,690, 1,560 and 900–1,350 cm^-1^ range of broad absorption peaks. This represents the results of the characteristic peaks of C=O, C=C and the broad O-H stretching, respectively. We also found that a large wide absorption peak could be observed at 3,000–3,800 cm^-1^ in each experimental group, which can be attributed to the broad O-H stretch caused by moisture associated with materials. The composite sample in shows a combination of MWCNT and HA peaks with peaks close to HA, which proves the successful combination of MWCNT and HA (Figure 1D).

MWCNT is nested-like, hollow cylindrical graphene structure inside. It has a strong absorption capacity in the near-infrared region. MWCNTs have a wider absorption spectrum compared to single-walled carbon nanotubes (SWCNTs), suggesting that MWCNTs can be activated by a larger spectrum of near-infrared light. And unlike SWCNT, MWCNTs can absorb more near-infrared radiation because they have a high specific surface area, and each particle in it has more electrons that can absorb light (Hirsch et al., 2003; Burke et al., 2009; Lin et al., 2015).

**FIGURE 2:**
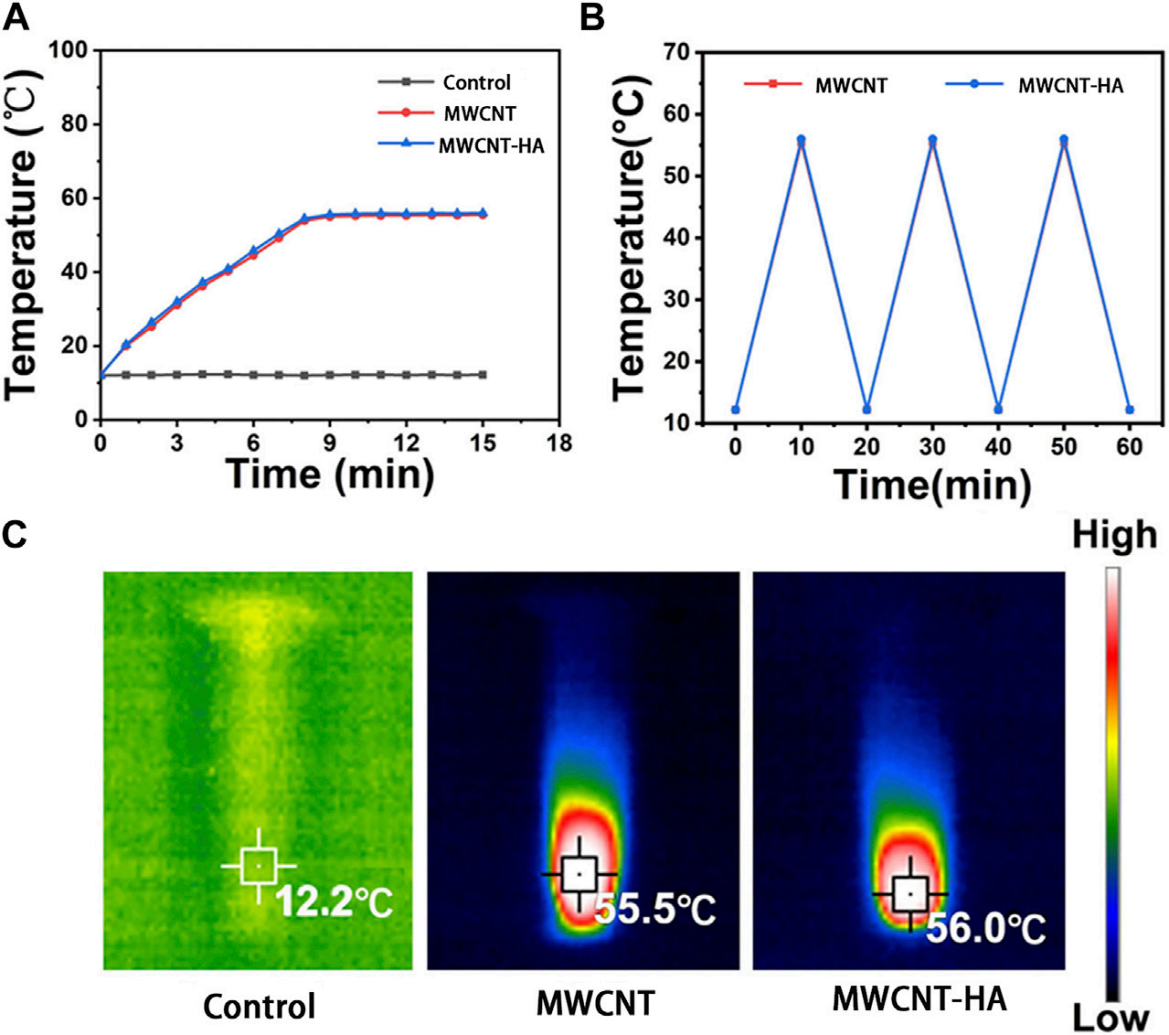
Characterization of MWCNTs and MWCNT-HAs. **(A)** Temperature rise curves of three groups of samples within 15 min**(B)** The terminal curves of MWCNTs and MWCNT-HAs after repeated laser irradiation **(C)** Infrared thermography images of samples under 808 nm laser irradiation for 5 min.

To verify the photothermal stability of MWCNTs and MWCNT-HA, we performed the experiments in Figure 2, and the results are shown. As shown in Figure 2A, the temperature of the blank control group was always maintained at 16°C without any increasing trend. In the MWCNT and MWCNT-HA groups, the temperature increased significantly in the 0–8 min interval, and the highest temperature reached 56°C after 8 min of irradiation. Both had a significant and similar temperature increase trend under the same irradiation conditions, and kept the highest temperature with time. This indicates that both of them have good photothermal conversion performance. As Figure 2B shown, we took 20 min as a node and found that there was no significant difference in the temperature rise trend between MWCNT and MWCNT-HA after three cycles, which indicated that both MWCNT and MWCNT-HA had excellent photothermal stability. In addition, in Figure 2C we can clearly observe that the maximum temperature of MWCNT and MWCNT-HA reached 56°C, and this temperature can be sufficient to cause irreversible cell damage. In summary, both MWCNT as well as MWCNT-HA have strong photothermal effects and good photothermal stability.

### 3.2 Effect of near-infrared light-responsive MWCNT and MWCNT-HA on cell proliferation *in vitro*

Tumor cells are a class of abnormal cells that have undergone genetic changes due to the action of tumorigenic factors and are unable to regulate the control of their own growth, in this case, this class of uncontrolled cells, they produce a continuous stimulation of normal cells, which leads to their proliferation, differentiation or death, resulting in abnormal cell proliferation (Shen et al., 2020; Yadav and Mohite, 2020). In contrast, malignant tumors are very fast-growing diseases that, once present, tend to metastasize and thus affect other organs and tissues of the body. The location of the tumor in the affected organs or tissues will produce substances that are harmful to the body, which in turn will cause the destruction of the nature and loss of function of the organs in the primary and metastatic sites, which will result in the appearance of some other diseases, and in serious cases, will be life-threatening (Juan and Xiaojing, 2019; Baltazar et al., 2020). In addition, malignant tumor cells are significantly different from normal cells in terms of metabolic and physiological characteristics. Due to their extremely fast and uncontrollable growth rate, the pathogenic ability of tumors is greatly enhanced. Therefore, the ability to effectively inhibit the growth and proliferation of tumor cells can be of great help in antitumor therapy (Shen et al., 2021).

OD value is an abbreviation for optical density. Absorbance refers to the use of the absorption spectrum unique to the substance to identify the substance or determine the content of the substance, and the detected object can absorb part of the light, and calculate the concentration of the detected substance through absorbance. Generally, the higher the concentration of the detected substance, the higher the value of absorbance (Mira et al., 2022).

To confirm the effect of near infrared laser (NIR) response to MWCNT and MWCNT-HA on CNE-1 cell proliferation, we determined the proliferation efficiency of CNE-1 cells after 24, 48, and 72 h of culture using the CCK-8 method.

**FIGURE 3:**
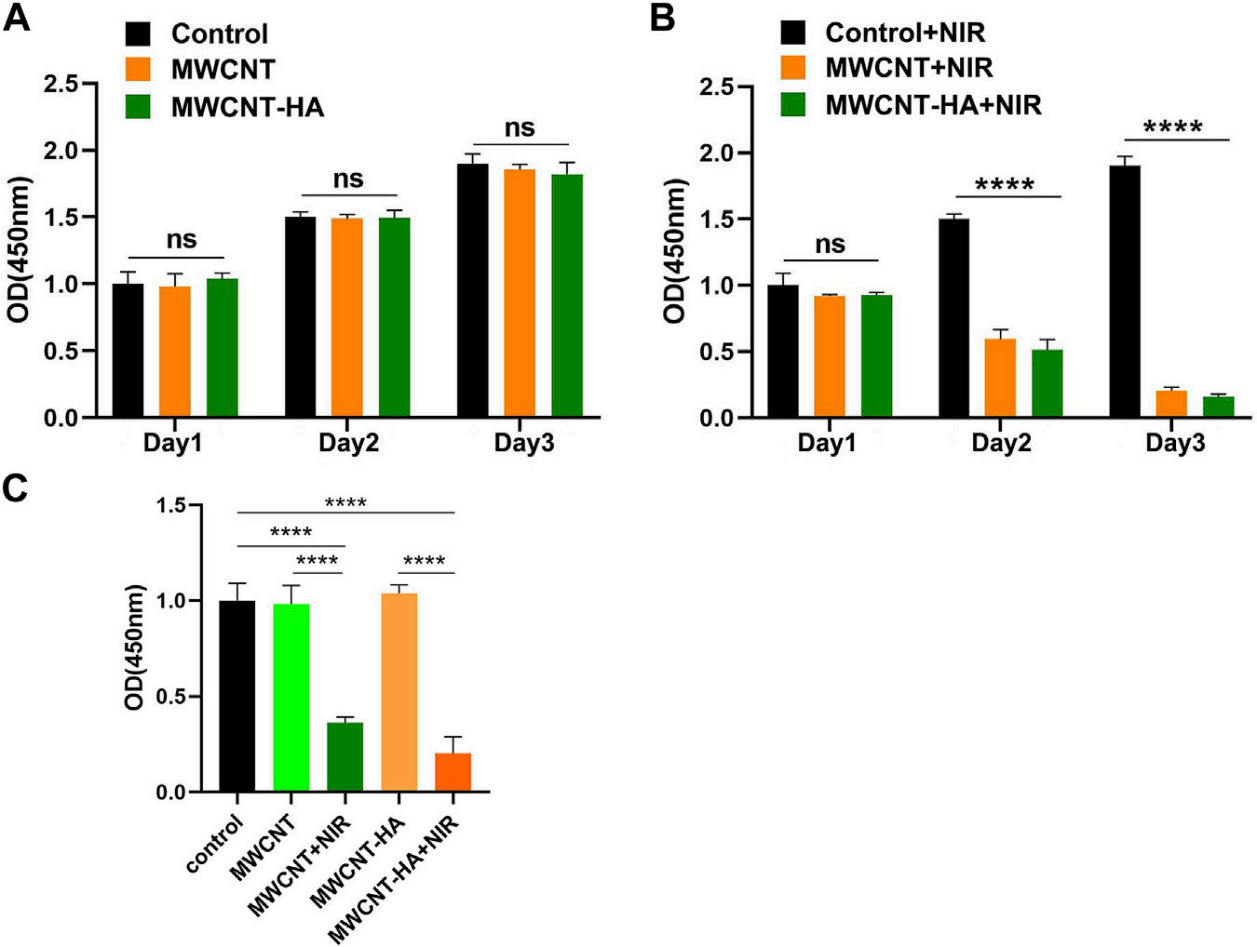
NIR-responsive MWCNT/MWCNT-HA inhibited proliferation of CNE-1 cells. **(A) (B)** Effects of NIR-responsive MWCNT/MWCNT-HA at different times on the proliferation of CNE-1 cells observed by CCK-8 method. **(C)**Overall OD values of each experimental group based on comprehensive 3-day experimental data. The mean and standard deviation (SD) of the data is shown. Statistical analysis: ns means no significance (*p* > 0.05), ****p < 0.0001.

As shown in Figures 3A,B, the mean and standard deviation (SD) of the data is shown. The mean and standard deviation (SD) of the data is shown in Figures 3A,B. In Figures 3A,B, we can find that in the experimental group without laser irradiation, the OD values of the 3 cells showed a significant increasing trend, and the growth trends of the MWCNT and MWCNT-HA groups on the first, second and third days were almost the same as the control group. After irradiation of the MWCNT and MWCNT-HA groups with NIR laser for 2 h, the cell proliferation rate showed a clear and gradual decrease over time. The OD values of the cells decreased significantly on the second day compared to the first day, and on the third day compared to the second day, their OD values were significantly smaller than the second day, indicating that MWCNT and MWCNT-HA significantly affected the cell proliferation under NIR laser irradiation. The combined situation of the cell proliferation experiment for 3 days was as follows Figure 3C shown. This series of results indicates that both MWCNT and MWCNT-HA responding to NIR laser effectively inhibited the proliferation of tumor cell.

**FIGURE 4:**
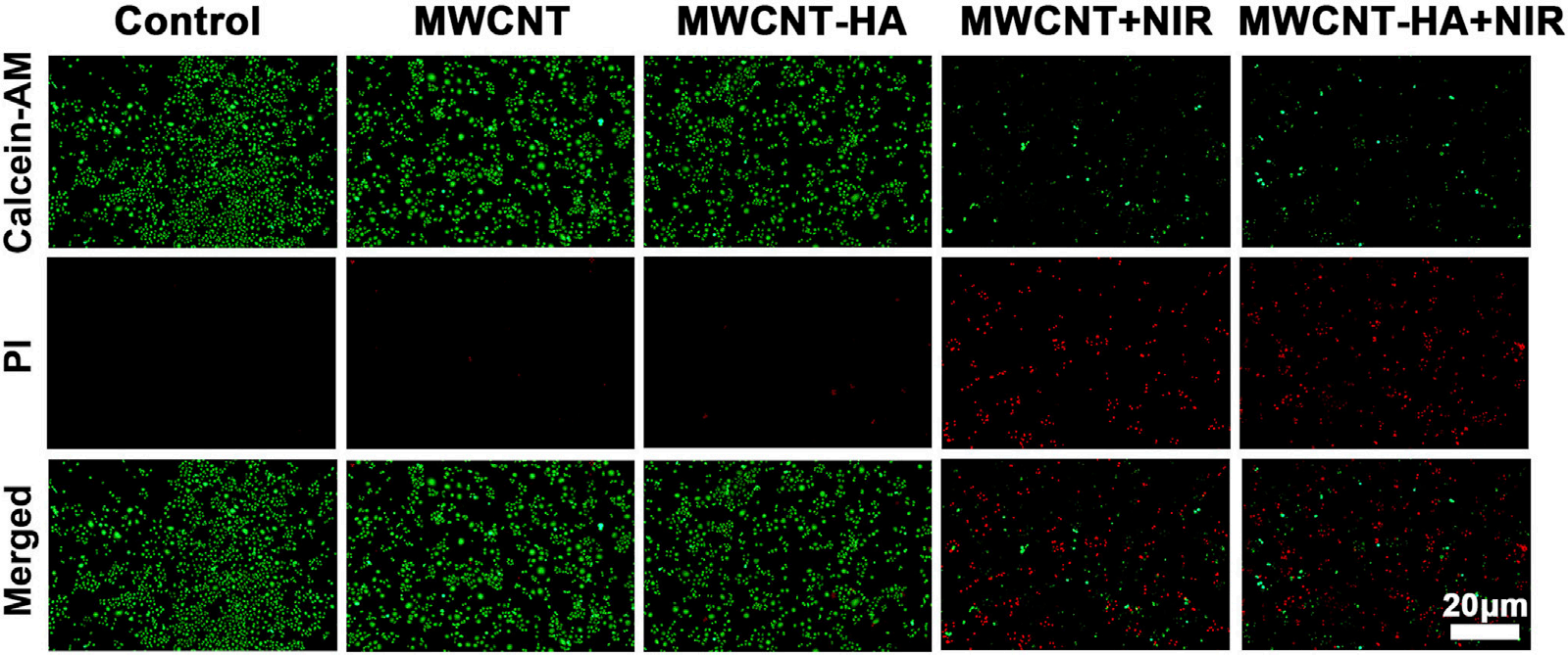
Fluorescent images of Live/Dead-stained CNE-1 cells after different treatments. Green: live cell staining, red: dead cell staining. Scale bars are 20 μm.

We also evaluated and verified the effect of MWCNT and MWCNT-HA on cell proliferation by performing live/dead cell staining. According to Figure 4, we found that the control, MWCNT-only group showed only green color with little red fluorescence, and the MWCNT-HA group showed a trace of red fluorescence, which was attributed to the programmed cell death itself. As we expected, the cells in the MWCNT + NIR group with MWCNT-HA + NIR group showed a large amount of red fluorescence. This indicates that both caused great killing of tumor cells under laser irradiation conditions. We can also see that the degree of cell killing in the MWCNT + NIR group and MWCNT-HA + NIR group is roughly similar, and both groups exhibit good anti-tumor ability.

**FIGURE 5:**
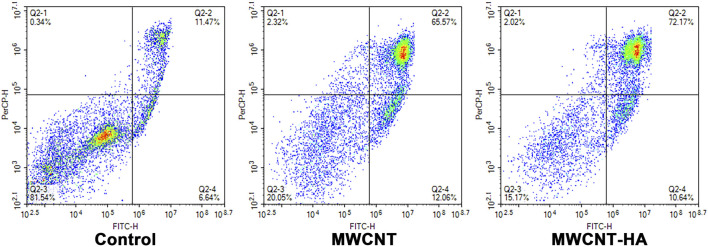
Flow cytometry analysis of CNE-1 cell apoptosis after MWCNT + NIR, MWCNT-HA + NIR treatment.

This was fully confirmed by subsequent flow cytometry analysis of AnnexinV FITC/PI staining (Figure 5), the maximum apoptosis rate of CNE-1 cells reached 77.63% and 82.81% in the MWCNT + NIR experimental group and MWCNT-HA + NIR experimental group, respectively, compared to the control group. This is because both MWCNT and MWCNT-HA composites have good photothermal conversion efficiency, so they caused a large number of tumor cell deaths under laser irradiation conditions. The results of flow cytometry analysis were also consistent with the results of Calcein/PI staining. These results proved that multi-walled carbon nanotubes assisted photothermal therapy achieved good therapeutic effects.

### 3.3 Effect of NIR-responsive MWCNT-HA on apoptosis *in vitro*

Apoptosis, also known as programmed cell death, is a genetically mediated, self-clearing process that occurs periodically in order to maintain a homeostatic balance between the rate of cell division and cell death *in vivo*, which generally removes cells that are functionally aged, not involved in the response or undergo abnormalities. However, due to mutations in certain genes, it may cause a misalignment of the apoptotic program and incomplete cellular self-clearance, and the resulting gradual accumulation leads to the development of various malignancies (D'Arcy, 2019; Obeng, 2021). Mitochondria are energy-producing structures in cells, which play a key role in the apoptotic pathway (Han et al., 2021). In turn, apoptosis is characterized by structural changes in the cell (D'Arcy, 2019). Mitochondrial dysfunction is one of the distinguishing features, which can lead to the development of many diseases, especially in cancer (Cho et al., 2020; Xue et al., 2020). In tumor cells, the mitochondrial apoptotic pathway is abnormally blocked and tumor cells develop mechanisms to evade apoptosis, allowing them to live forever without self-clearance. In contrast, Caspase-3 and Bcl-2 are the protein factors associated with the apoptotic pathway in mitochondria (Hainaut and Plymoth, 2013; Ward et al., 2018; Wong et al., 2019).

To investigate the mechanism by which multi-walled carbon nanotubes promote apoptosis induction, we observed the factor staining of CNE-1 cells using inverted fluorescence microscopy after 72 h of cell culture and explored the expression levels of apoptotic factors Caspase-3 and Bcl-2 by statistical analysis using SPSS software.

**FIGURE 6:**
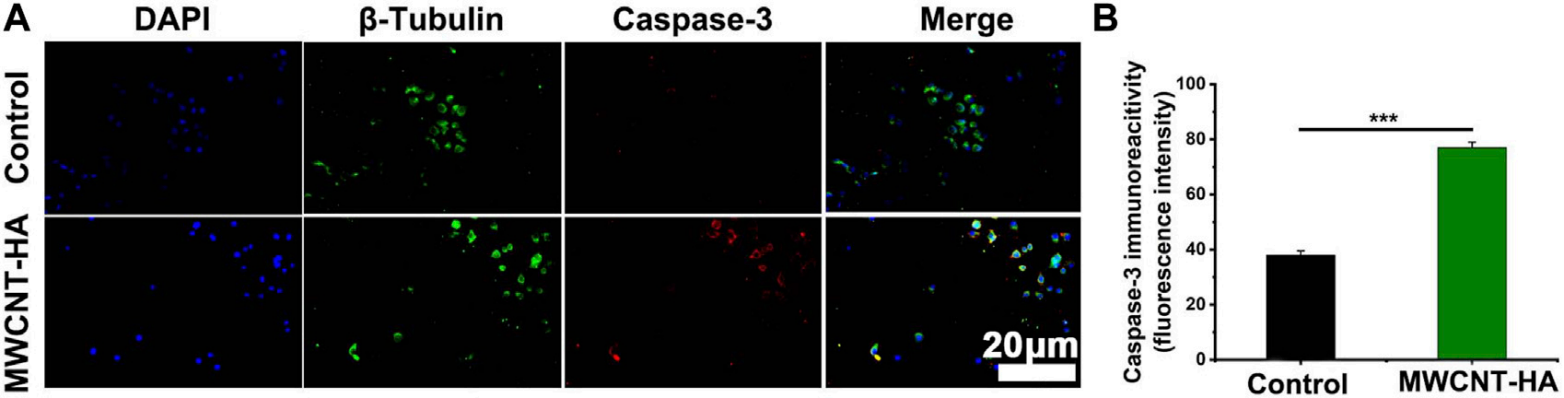
NIR-responsive MWCNT-HA promoted the expression level of Caspase-3 protein in CNE-1 cells **(A, B)**. Fluorescence inverted microscopy immunofluorescence analysis of Caspase-3 protein expression and its semi-quantification analysis. Scale bars are 20 μm. The mean and standard deviation (SD) of the data is shown. Statistical analysis: ***p < 0.001.

In Figures 6A,B, we found that the expression level of Caspase-3 factor was increased to about 80% in MWCNT-HA group compared with the control group.

**FIGURE 7:**
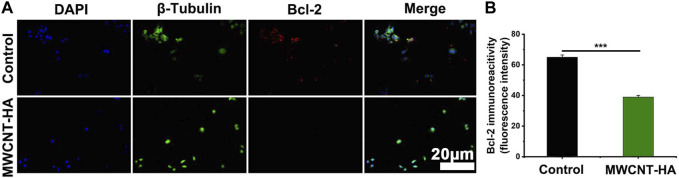
NIR-responsive MWCNT-HA promoted the expression level of Bcl-2 protein in CNE-1 cells **(A, B)**. Fluorescence inverted microscopy immunofluorescence analysis of Bcl-2 protein expression and its semi-quantification analysis. Scale bars are 20 μm. The mean and standard deviation (SD) of the data is shown. Statistical analysis: ***p < 0.001.

Meanwhile, we also found that the expression of the anti-apoptotic factor Bcl-2 was significantly downregulated in the MWCNT-HA group compared to the control group, with a decrease of up to 26% (Figures 7A,B). This phenomenon suggests that under the conditions of NIR laser irradiation, MWCNT-HA can lead to cell death by inhibiting the mitochondrial pathway of CNE-1.

### 3.4 Effect of NIR-responsive MWCNT-HA on cell morphology *in vitro*

Tumor cells also differ significantly from normal cells as well as tumor cells in terms of size and morphological characteristics. Cell morphology can serve as a way of reading information that is highly relevant to the molecular basis, and the morphological state of cells is closely related to their basic physiological characteristics (Yin et al., 2013; Wu et al., 2015). Therefore, under specific conditions, cell morphology is a newly emerging and easier to perform measurement compared to other experiments (Wu et al., 2020).

**FIGURE 8:**
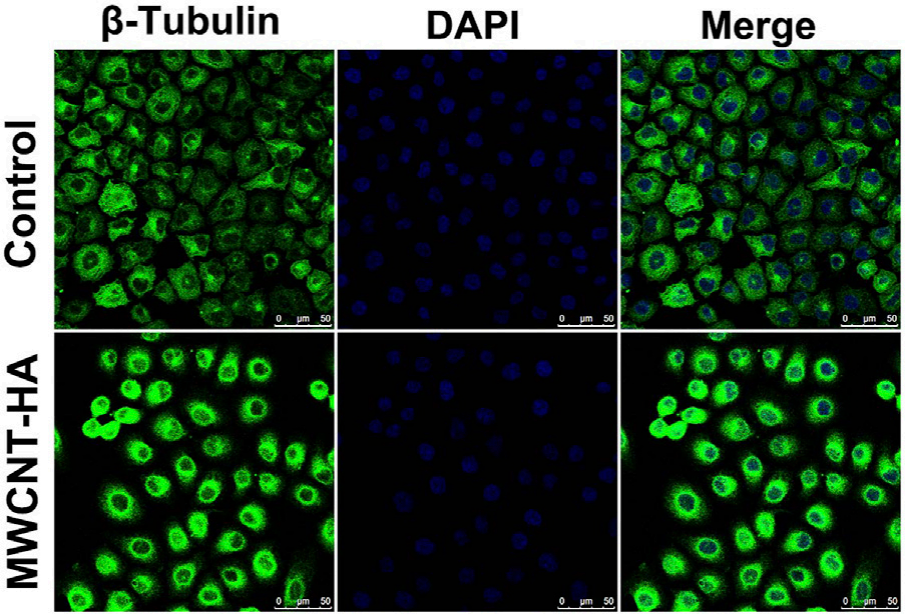
Confocal Fluorescence Microscopy Used for Morphological Analysis of CNE-1 Cells. Scale bars are 50 μm.

In order to investigate the effect of multi-walled carbon nanotubes on cell morphology in photothermal treatment, we observed the morphological changes of two groups of cells using confocal fluorescence microscopy after the cells were cultured and treated for 72 h. The morphology of normal CNE-1 cells was spindle-shaped cells and irregular in shape, As shown in Figure 8, the cells were attached to each other and well organized. In the MWCNT-HA + NIR treated group, we found that the cell morphology changed, with fewer intercellular connections and cell crumpling, and the morphology changed to spherical. We also found that the number of cells in the MWCNT-HA treated group was reduced compared to the control group, which was due to the apoptotic floating of cells after laser irradiation treatment, and some of the dead cells were washed away by PBS during the pre-treatment resulting in a reduced number of cells. These some results suggest that multi-walled carbon nanotubes-hyaluronic acid can cause significant morphological changes in the cells after NIR laser irradiation.

## 4 Discussion

Studies have shown that modified multi-walled carbon nanotubes interfere with the growth and development of tumor cells by inhibiting cell proliferation, enhancing cytotoxicity, inducing apoptosis, and blocking the cell cycle, and can be used as drug carriers to piggyback chemotherapeutic drugs to kill tumors more effectively (González-Lavado et al., 2018; González-Lavado et al., 2019). The unique hollow structure of MWCNT can give full play to the high thermal conductivity of the material itself under near-infrared laser irradiation, convert light energy into heat energy, affect the mitochondrial apoptosis pathway of tumor cells, upregulate the expression of the apoptosis factor Caspase-3 in the upper reaches of the mitochondrial apoptosis pathway, and then affect the expression of the anti-apoptotic factor Bcl-2 located downstream along the pathway, resulting in apoptosis of CNE-1 cells.

In this study, we obtained MWCNT-HA composites with good biocompatibility and photothermal conversion properties by using MWCNT and by purifying MWCNT, treating MWCNT with acid and activating it, and exploiting the thermal conductivity of the material by near-infrared laser irradiation. The final experimental results also show that the photothermal properties of MWCNT and MWCNT-HA can be applied in tumor photothermal therapy, and provide some experimental basis for the synthesis of engineered MWCNT to reduce the riskiness of MWCNT.

## 5 Conclusion

In summary, we successfully prepared MWCNT-HA composites and deeply investigated the killing effect of both materials on tumor cells under the influence of NIR laser. This study can provide a novel composite material for photothermal therapy of nasopharyngeal cancer. The results of thermographic correlation mapping showed that the multi-walled carbon nanotubes as well as the prepared MWCNT-HA possessed near-infrared photothermal and maintained good photothermal stability after multiple laser cycles of irradiation. Moreover, the cell viability of the MWCNT group and the MWCNT-HA group decreased by about 80% in the 3 days after the NIR light irradiation treatment. Cell inverted fluorescence microscopy analysis showed that MWCNT-HA significantly upregulated the expression of Caspase-3 by nearly 1-fold and downregulated the expression of Bcl-2 by about 26% after NIR irradiation. It was also confirmed after the observation of cell morphology by confocal microscopy that the treated MWCNT-HA had a significant effect on the morphology of CNE-1 cells and sufficiently promoted the apoptosis of the cells. This study provides useful information for multi-walled carbon nanotubes as efficient auxiliary nanomaterials for photothermal therapy.

## Data Availability

The original contributions presented in the study are included in the article/supplementary material, further inquiries can be directed to the corresponding author.
